# Yolk sac tumor of vagina: a rare cause of vaginal bleeding in adolescents - a case report

**DOI:** 10.11604/pamj.2020.37.169.20949

**Published:** 2020-10-16

**Authors:** Meriem Elbaz, Rabiy El Qadiry, Karima Fouraiji, Hicham Jalal, Jamila Elhoudzi

**Affiliations:** 1 Pediatric Hematology and Oncology Department, Mohamed VI University Hospital, Marrakech, Morocco,; 2 Pediatric Surgery Department, Mohamed VI University Hospital, Marrakech, Morocco,; 3 Pediatric Radiology Department, Mohamed VI University Hospital, Marrakech, Morocco

**Keywords:** Vaginal bleeding, child, yolk sac tumor, serum alpha fetoprotein negative

## Abstract

Yolk sac tumor (YST) is one of the malignant germ-cell tumors (MGCT) that usually occurs in the ovaries and testes of young patients. Its occurrence in the vagina is extremely rare. We present a rare case of extragonadal YST occurring in the vaginal region. A 12-year-old girl, presented with vaginal bleeding and pain in the perineal region. Physical exam identified a limited pelvic mass, 5 x 4cm in size. Abdominal ultrasound and magnetic resonance imaging (MRI) showed a heterogeneous mass in cervico-vaginal region. The patient was taken to the surgery, where an excisional biopsy was obtained. The diagnosis of YST is confirmed by histopathology and immunohistochemistry studies. However, the tumor marker (alpha fetoprotein and BHCG) was normal. The patient was treated according to the French TGM-95 protocol. Surgery was done after chemotherapy, hysterectomy in front of the cervical invasion, with a good decline at 2 years of end of treatment.

## Introduction

Malignant germ-cell tumors (MGCT) are rare in childhood and represent less than 5% of all pediatric cancers [1]. Yolk sac tumor is one of the most common histological subtypes of MGCT in children that usually occurs in the ovaries and testes of young patients before 3 years of age. Its occurrence in the vagina is extremely rare. Early detection and therapy is important because of its aggressive nature and good response to chemotherapy. We present a rare case of extragonadal yolk sac tumor occurring in the vaginal region of a 12-year-old girl.

## Patient and observation

**Figure 1:**
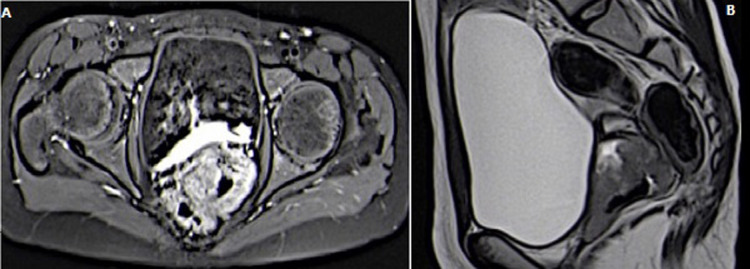
A) T1 FAT SAT with contrast MRI shows mass occupying the vagina with heterogeneous enhancement; B) T2 without contrast showing the vaginal mass with cervical involvement and respect of posterior side of the bladder

A 12-year-old girl with no previous medical history presented with vaginal bleeding and pain in the perineal region reporting the onset of the symptoms three months before. A physical examination identified a pelvic mass, 5 x 4cm in size, not painful. The pervaginal exam was difficult given the age of the girl and the absence of suitable equipment. Radiological studies (ultrasound and MRI) showed a heterogeneous mass measuring 5.1 x 6.2 x 6.5cm in cervico-vaginal region (Figure 1). There were no liver or lung metastases. The tumors markers (serum alpha-fetoprotein, beta-human chorionic gonadotropin) were undetectable.

**Figure 2:**
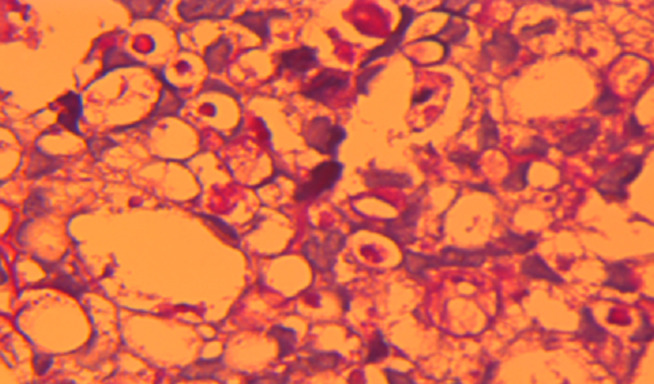
numerous intracellular and extracellular periodic acid-Schiff positive diastase, (periodic-acid-Schiff x400)

**Figure 3:**
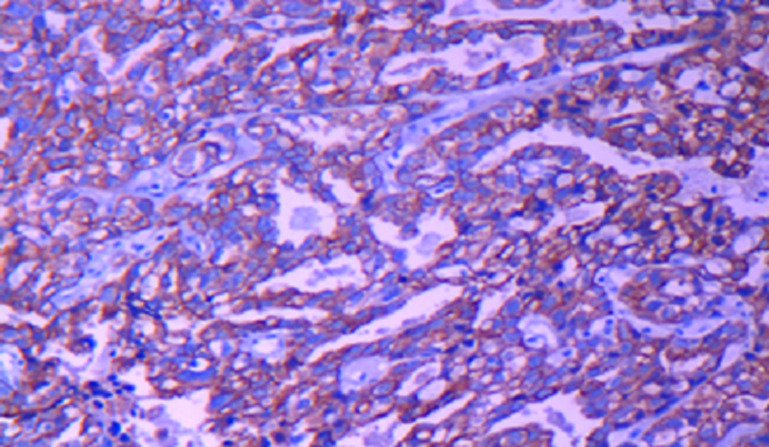
immunohistochemistry stain shows focal positivity of AFP++ and cytokeratin (AE1/AE3)+

**Figure 4:**
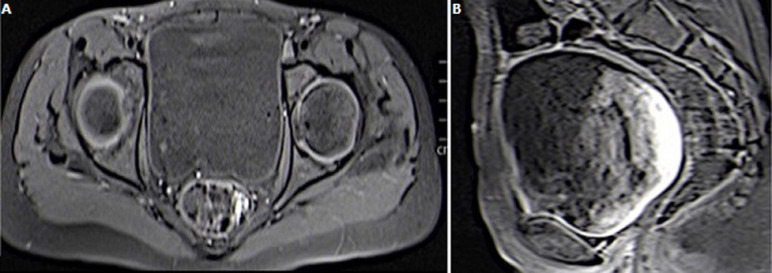
MRI 3 months after the surgery showed absence of centropelvic mass

The patient was taken to the surgery, where an excisional biopsy was obtained. Microscopic examination showed neoplastic cells arranged in a reticular growth pattern with glomerular-like structures composed of a central blood vessel enveloped by atypical large pleomorphic cells with prominent nucleoli (Schiller-Duval bodies) and numerous intracellular and extracellular periodic acid-Schiff positive diastase (Figure 2). Immuno-histochemical studies revealed that the tumor cells were focally positive for alpha feto-protein (AFP) and cytokeratin (AE1/AE3) (Figure 3) and negative for skeletal muscle markers (desmin and myogenin), this result was confirmed by a second pathology look. Histopathologic examination and immunohistochemical studies support the diagnosis of yolk sac tumor, however, the AFP serum was negative. After many discussions with surgeon and review of pathological exam we decided to treat as YST. The patient received six courses of etoposide, ifosfamide and cisplatin according to the French TGM-95 protocol. The MRI monitoring after this neo-adjuvant chemotherapy showed a decrease in tumor volume by more than 50% but with cervical invasion a hysterectomy was indicated. There is no evidence of recurrence at two years of follow-up (Figure 4).

## Discussion

Vaginal bleeding in young girls poses a challenge. The etiology of vaginal bleeding in young girls varies widely from idiopathic bleeding to sexual abuse and malignant tumors. Although genital tumors are very rare in childhood it is important that they are ruled out when a girl presents with unexplained vaginal bleeding. A genital examination, which can be used to diagnose most pediatric gynecological problems is recommended in all cases [2]. MGCT arising primarily from the vagina are extremely rare comprising from 3 to 8% of all malignant germ-cell tumors. Endodermal sinus tumor (EST) or yolk sac tumor (YST) forms the most common histological subtype of GCT in children that exclusively involves children less than 3 years of age. In children, the tumors are frequently located in the sacro-coccygeal region, testis and ovary. The extra-gonadal location of YST could be the result of germ cells “lost” during their migration from the yolk sac along the dorsal wall of the embryo to the gonadal folds [3].

The clinical presentation includes a history of bloody vaginal discharge, often accompanied by a polypoid mass protruding from the vagina which was not the case in our patient. Most common differential diagnosis is botyroides sarcoma differentiated by its characteristic grape like appearance and histopathology [4]. The use of serum AFP as tumor marker has been well established for patients with YST. Some cases of YST without elevated serum AFP have also been reported, the histological patterns of these tumors tend to be atypical and the lack of serum AFP elevation may be correlated with the lack of AFP expression in the majority of tumor cells that formed a solid pattern and was hence, not associated with elevated serum AFP [5,6]. In our case the serum AFP was undetectable, the diagnosis was based on histological pattern characteristic, presence of diastase resistant PAS stain positive hyaline globules. The immunohistochemical stains for alpha-fetoprotein strongly react with the tumor cells and thus confirm the diagnosis of YST.

Most of the cases of vaginal YST reported in the literature are limited to the vagina, only a few of them report involvement of the cervix [7], which was the case in our patient and because of that a radical hysterectomy was performed after parental consent. Partial vaginectomy with combination chemotherapy is the most recommended line of treatment [8,9]. To allow preservation of sexual and reproductive function, chemotherapy as a sole modality of treatment for YST should be considered [10]. In our case, patient received six courses of VIP (ifosfamid, etoposide and cisplatin) which reduced the tumor size by 50% then underwent surgery. The cervical involvement and the late of diagnosis are the main reasons for invasive surgery in our case. There is no evidence of recurrence at two years of follow-up.

## Conclusion

YST in the vagina is very rare and may not be recognized early. Final diagnosis is obtained by histological examination and AFP levels, which are also helpful during follow-up. Recommended treatment includes neo-adjuvant chemotherapy and conservative surgery because together they improve the prognosis and preserve both reproductive and sexual function.
